# Subchronic Toxicities of HZ1006, a Hydroxamate-Based Histone Deacetylase Inhibitor, in Beagle Dogs and Sprague-Dawley Rats

**DOI:** 10.3390/ijerph13121190

**Published:** 2016-11-30

**Authors:** Xiaofang Zhang, Xiaodong Zhang, Bojun Yuan, Lijun Ren, Tianbao Zhang, Guocai Lu

**Affiliations:** Center for Evaluation of Drug Safety, Second Military Medical University, 800 Xiang Yin Road, Shanghai 200433, China; haidaofang1980@163.com (X.f.Z.); xiaodongzhang@163.com (X.d.Z.); ybj74386@aliyun.com (B.Y.); renlijun@163.com (L.R.)

**Keywords:** Hb-HDACIs, HZ1006, safety assessment, NOAEL

## Abstract

Histone deacetylase inhibitors (HDACIs), such as vorinostat and panobinostat, have been shown to have active effects on many hematologic malignancies, including multiple myeloma and cutaneous T-cell lymphoma. Hydroxamate-based (Hb) HDACIs have very good toxicity profiles and are currently being tested in phases I and II clinical trials with promising results in selected neoplasms, such as bladder carcinoma. One of the Hb-HDACIs, HZ1006, has been demonstrated to be a promising drug for clinical use. The aim of our study was to determine the possible target of toxicity and to identify a non-toxic dose of HZ1006 for clinical use. In our studies, the repeated dosage toxicity of HZ1006 in Beagle dogs and Sprague Dawley (SD) rats was identified. Dogs and rats received HZ1006 orally (0–80 and 0–120 mg/kg/day, respectively) on a continuous daily dosing agenda for 28 days following a 14-day dosage-free period. HZ1006’s NOAEL (No Observed Adverse Effect Level) by daily oral administration for dogs and rats was 5 mg/kg and 60 mg/kg, respectively, and the minimum toxic dose was 20 and 120 mg/kg, respectively. All the side effects indicated that the digestive tract, the male reproductive tract, the respiratory tract and the hematological systems might be HZ1006 toxic targets in humans. HZ1006 could be a good candidate or a safe succedaneum to other existing HDACIs for the treatment of some solid tumor and hematologic malignancies.

## 1. Introduction

The use of histone deacetylases inhibitors (HDACIs) as anti-cancer agents for hematological malignancies and solid tumors has been actively explored. During the past several years, many HDACIs have entered pre-clinical or clinical research as anti-cancer agents with satisfying results. Vorinostat was the first HDAC inhibitor approved by the US Food and Drug Administration for treating cutaneous T-cell lymphoma patients. Out of these, more than eight novel hydroxamic acid based HDACIs, i.e., belinostat, romidepsin, abexinostat, SB939, resminostat, givinostat, quisinostat, pentobinostat, and CUDC-101 are in clinical trials. Romidepsin was approved for the treatment of cutaneous T-cell lymphoma (CTCL) by FDA in 2009. SB939 is an orally available, competitive histone deacetylase (HDAC) inhibitor selective for class I, II and IV histone and was conducted a phase I trial in patients with advanced solid tumours by National Cancer Institute of Canada (NCIC).

In addition to the therapeutic effect, the particularly important thing to consider is a drug’s safety or toxicity. It is found that the primary drug-related side effects of vorinostat after 26 weeks orally dosed daily with a 4-week recovery are decreased food consumption, weight loss, and hematologic changes on rats (controls, 20, 50, or 150 mg/kg/day) and primarily gastrointestinal lesion on dogs (controls, 60, 80, or 100/125/160 mg/kg/day) respectively. The no-observed adverse effect level is not established in rats and 60 mg/kg/day (* 6-fold maximum recommended human dose) in dogs, toxicities are reversible and can be monitored in the clinic. ST and T wave changes, QTc prolongation, and/or torsade de pointes have been reported with SAHA, LBH589B, and MS-275 [1].

Shah et al. [2] reported on the cardio-toxicity of the histone deacetylase inhibitor romidepsin (depsipeptide, FK228) administered weekly 3 q28, in 15 patients with metastatic neuroendocrine tumors. The study was terminated early due to a high number of ‘serious cardiac adverse events’ comprising of one sudden death, asymptomatic grade 2 ventricular tachycardia in two patients, and prolonged QTc interval in three patients, all probably related to romidepsin. Another example of adverse effects of romidepsin was a phase I trial conducted by Marshall et al. [3] The MTD of depsipeptide given on a day-1 and -5 schedule every 21 days is 17.8 mg/m^2^. The dose-limiting toxicity are fatigue, nausea, vomiting, and transient thrombocytopenia and neutropenia. Whereas cardiac toxicity was anticipated based on preclinical data, there was no evidence of myocardial damage. However, reversible ECG changes with ST/T wave flattening were regularly observed.

In a study by Razak et al. [4] administration of SB939 in a daily 5 schedule in patients with advanced solid tumors resulted in toxic effects that included fatigue, nausea, vomiting, anorexia and diarrhea. These toxicities, however, were non-life threatening, and were effectively addressed by dosing delay and dose reduction. While QTc elevations were documented, none were grade 3. More data on the frequency of QTc changes will be known following phase II trials where monitoring during cycle 1 will be undertaken.

**Figure 1:**
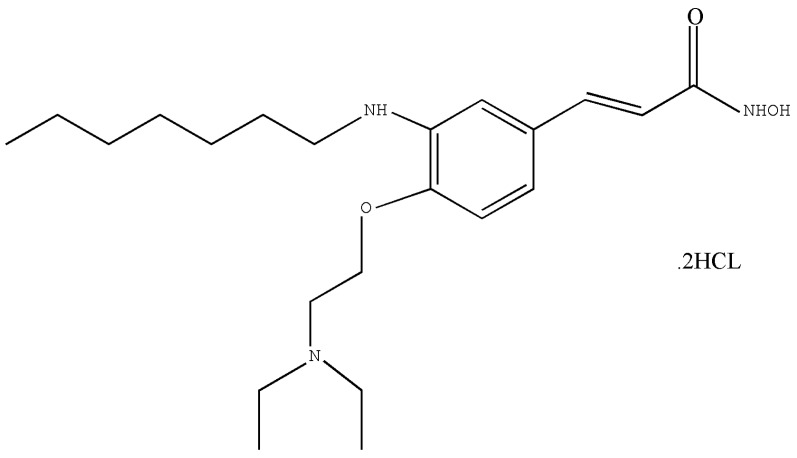
Chemical structure of HZ1006.

According to the shortcomings and safety issues of HDACIs for cancer treatments, opportunities still exist for the exploitation of novel compounds with better safety and/or tolerability characteristics. HZ1006 (the chemical structure is shown in Figure 1 and its molecular formula and weight are C_22_H_37_N_3_O_3_·2HCl and 464.62, respectively), a newly synthesized compound and another hydroxamate-based HDACIs, has been developed by the Zhejiang Haizheng Pharmaceutical Co., Ltd. (Zhejiang, China). It has demonstrated lower acute toxicity for dogs and rats than other HDACIs such as SAHA (data unpublished). The manufacturer of HZ1006 is applying for phase I clinical trial in China now. The aim of our study was to define the adverse reaction of HZ1006 in Beagle dogs and SD rats following repeated administration and to provide information for upcoming clinical use.

## 2. Materials and Methods

### 2.1. Test Article

HZ1006 was provided by the sponsor and is an off-white powder. It should be stored at 2–8 °C and protected from light. When needed, the chemical compound was dissolved and diluted with demineralized water one day before administration. The concentration and stability of HZ1006 after preparation were tested in accordance with the requirements of Good Laboratory Practice (GLP) for non-clinical research principles (cFDA, 2003).

### 2.2. Animals

Forty (20 male and 20 female) purebred Beagle dogs aged 8–10 months, whose body weights ranged from 7 to 10 kg as used in repeated dosage toxicity study on non-rodents, were purchased from the Shanghai Xingang Laboratorial Animal Co., Ltd. (Shanghai, China). All the animals were housed one per cage (length × width × height: 800 × 650 × 740 mm^3^) in a room where the temperature remained between 18 and 28 °C, the relative humidity was 40%–70%, lighting was 10–12 h per day and air ventilation was 10 cycles per hour.

Eighty (40 male and 40 female) healthy Sprague-Dawley (SD) rats aged 6–8 weeks, whose body weights ranged from 160 to 200 g, were purchased from the Super-B&K Laboratory Animal Corp., Ltd. (Shanghai, China). Every cage (length × width × height: 300 × 400 × 210 mm^3^) housed five rats. All the animals were housed in a room where the temperature remained between 20 and 26 °C, the relative humidity was 40%–70%, lighting was 12 h per day, and air ventilation was 20 cycles per hour.

All the dogs and rats used in the studies had free access to feed and water.

The studies followed GLP principles (2003) and the Guideline for the Testing of Chemicals in Chronic Toxicity studies ([H]GPT2-1, 2005) enacted by the Food and Drug Administration of China. The guidelines for institutional animal care were followed when the rats and dogs were bred and dissected, and the local Institutional Committee of Second Military Medical University (SMMU) approved the study protocols (protocol 2012-06-13).

### 2.3. Doses and Treatment Plans

#### 2.3.1. Subchronic Toxicity Test in Beagle Dogs

HZ1006 was administered orally to dogs (four/sex/group) at doses of 80, 40, 20, 5 and 0 (control) mg/kg/day, and the dosage volume was 2 mL/kg. The dogs received test doses with the exact amounts on a weekly basis based on current body weights. Three dogs died during the study, including ♀6 on d8, ♀8 on d10 in the 80 mg/kg dose group and ♀28 on d16 in the 40 mg/kg dose group. The other dogs in the above groups were dissected on the planned days. For the other groups, half of the dogs were anatomized on d28 and the remaining half on d42.

#### 2.3.2. Subchronic Toxicity Test in SD Rats

The rats that were judged suitable for the subchronic toxicity study were randomly divided into four groups (10 rats/sex/group). The rats received the test sample by gavage once daily for 28 days. HZ1006 was administered to the rats at doses of 120, 60, 20 and 0 (control) mg/kg, and the dosage volume was 5 mL/kg. Half of the 80 rats (half male and half female) were selected randomly according to body weight and dissected at d28. The remaining rats were dissected at d42.

### 2.4. Clinical Symptoms Observed on Dogs and Rats in Repeated Dose Toxicity Studies

Clinical symptoms and mortality were examined at least twice daily during the 42-day period, which included any changes in reaction to treatment, behavior, illness, food consumption, and body weight. Breathing rates, rectal temperature and pupil changes in the Beagle dogs were assessed once every 2 weeks.

### 2.5. Clinical Examination Parameters

The following indexes were examined for the dogs used in the study, twice during the pre-dosage period and on d14, d28 and d42. The following parameters were evaluated on d28 and d42 for the rats in the study:

#### 2.5.1. Electrocardiogram (ECG)

The parameters of the dogs’ ECG included the Heart rate, Time at R, P Duration, P-R interval, T Duration, QRS Durations, Q-T Intervals and ST Segment, which were examined by an ECG6511 ECG instrument (manufactured by Shanghai Kohden Medical Electronic Instrument Corp., Shanghai, China) on d0 (twice), d14, d28 and d42 using the II limb lead method. According to the requirements of the guidelines of China, the rats did not receive ECGs.

#### 2.5.2. Hematology

Blood samples of the animals were collected in tubes containing ethylenediamine tetraacetic acid (EDTA) and tested using an ADVIA2120 Hematology Autoanalyzer (Bayer, Leverkusen, Germany). The following indexes were examined: hemoglobin concentration (Hb), red blood cell count (RBC),mean corpuscular volume (MCV), hematocrit (HCT), mean corpuscular hemoglobin concentration (MCHC), mean corpuscular hemoglobin (MCH), white blood cell count (WBC), platelet count (Plt), WBC differential counts [neutrophilcyte (Neu), eosinophilic granulocyte (Eos), basophilic leukocyte (Bas), lymphocyte (Lym), and monocyte (Mon)] and reticulocytes (Ret). The serum coagulation times of the blood samples (1.8-mL samples were mixed with 0.2 mL sodium citrate), including prothrombin time (PT), fibrinogen (FIB), activated partial thromboplastin time (APTT), and thrombin time (TT), were tested using an MC10 plus Blood Coagulation Analyzer from Shanghai San Jose Medical Products Co., Ltd. (Shanghai, China).

#### 2.5.3. Serum Biochemistry

The animal blood samples were collected in the tubes containing the coagulator and centrifuged at 500 *g* for 10 min; the supernatant was then collected into a new tube. The serum biochemistry indexes included the following: alanine aminotransferase (ALT), alkaline phosphatase (ALP), aspartate aminotransferase (AST), blood urea (BU), lactate dehydrogenase (LDH), total bilirubin (TBIL), creatinine (Crea), albumin (ALB), total protein (TP), triglycerides (TG), total cholesterol (TCH), γ-glutamyltranspeptidase (g-GT), glucose (GLU), creatine phosphokinase (CPK), uric acid (UA), phosphorus (P), and calcium (Ca^2+^). The above parameters were analyzed using an Automatic Analyzer 7080 provided by Hitachi High-Technologies Corp. (Tokyo, Japan). An Na/K/Cl Analyzer EasyLyte PLUS from Medica Corp. (Bedford, MA, USA), was used to determine the levels of chloride (Cl^−^), sodium (Na^+^) and potassium (K^+^) in the blood samples.

#### 2.5.4. Urinalysis

A Micro AUTION MA-4260 Autoanalyzer from Arkray Global Business, Inc. (kyoto, Japan), was used to analyze the animal urine samples. Parameters such as pH, specific gravity, nitrites, leukocytes, glucose, protein, urobilinogen, ketones, occult blood, bilirubin, and hemoglobin were tested.

#### 2.5.5. Bone Marrow Examination

Bone marrow samples were obtained according to the methods reported previously [5]. We classified the bone marrow cells by immersion objective.

#### 2.5.6. Histopathology

After euthanasia, the following organs or tissues were isolated from the dogs and rats: skin, abnormal lesions, spleen, mammary glands, jejunum, pancreas, ileum, duodenum, stomach, colon, cecum, lymph nodes, mesenteric salivary glands, liver, submandibular lymph nodes, thymus, sternum, lungs, heart, esophagus, trachea, tongue, thyroid with parathyroid gland, sciatic nerve, aorta, femurs, skeletal muscle, pituitary gland, brain, kidneys, eyes, urinary bladder, adrenal glands, uterus, ovaries, testes, epididymis and prostate. The weights of the animal organs, such as the liver, spleen, heart, thymus, brain, lungs, adrenal glands, kidneys, uterus, ovaries, epididymis and testes, were tested using an electronic balance. The organs or tissues were fixed using 10% neutral buffered formalin and were then gradient dehydrated, embedded, sectioned, and stained with routine hematoxylin-eosin prior to histopathological observations.

The parameters mentioned above were selected according to the requirements in the Guidance for Chronic Toxicity studies Techniques for Chemicals ([H]GPT2-1, 2005) enacted by the Food and Drug Administration of China, and the detailed procedures were reported by Yu et al. [5].

### 2.6. Toxicokinetics

The dogs’ blood samples were obtained on the 1st day, the 14th day and the last day (d28) of dosage for plasma HZ1006 content analyses at 0 (1st day only), 1/6, 0.5, 1, 2, 4, 8, 12 and 24 h after dosing, and the blood samples of the rats were acquired on the 1st day and 28th day at the same time as those of the dogs, which were handled and analyzed according to the procedure reported by Zhang et al. [6].

### 2.7. Statistical Analysis

Parametric one-way analysis using the F-test (ANOVA, two-sided) and repetitive measure analysis of variance (ANOVA) were used to analyze the data with a professional statistical software package (Statistical Product and Service Solutions (SPSS) v16.0, IBM, Inc., Armonk, NY, USA) for dogs and rats respectively. NPar Kruskal–Wallis test was used to analyze urinalysis data.

## 3. Results

### 3.1. Subchronic Toxicology Study in Dogs

#### 3.1.1. Clinical Observation

The main clinical symptoms in the animals that were indicative of HZ1006 toxicity included loss of appetite and vomiting after eating in the 20-mg/kg dose group after administration. In the 40- and 80-mg/kg dose groups, the dogs’ main side effects following administration included partial to complete anorexia, repeated vomiting, inability to stand, diarrhea, decreased activity, fremitus, drooling, swollen limbs, deep and fast breathing and skin flushing. These symptoms reversed when the administration was stopped, except in dog ♀6 and ♀8 in the 80-mg/kg dose group, which died on d8 and d10, respectively, and dog ♀28, which belonged to the 40-mg/kg dose group and died on d16. Other parameters had no apparent changes when compared with the drug-free group (data on the frequencies of vomiting and diarrhea of the dogs are shown in Table 1).

#### 3.1.2. Hematology

The dogs in the 20-, 40-, and 80-mg/kg dose group showed significantly reduced RBC, HGB, and Hct on d14 and d28 when compared with d0 or the control group. The dogs belonging to the 40- and 80-mg/kg dose groups showed reduced RET on d14 and d28 when compared with d0 or the control group, especially for ♀6 and ♀28 animals, which died on d8 and d16, respectively (0.14% and 0.04%, respectively). The dogs belonging to the 20-, 40-, and 80-mg/kg dose groups showed significantly elevated monocyte % or a tendency toward elevation on d14 and d28 when compared with d0 or the dose-free group. The dogs belonging to the 40- and 80-mg/kg dose groups indicated significantly higher APTT and FIB on d14, and the 80-mg/kg dose dogs showed significantly higher FIB on d28 when compared with d0 or the control group, especially for ♀6 and ♀28 animals, which died on d8 and d16, respectively. The parameters mentioned above recovered after the 14-day drug-free period. There were no obvious changes observed in the other parameters studied (only data with statistical significance are listed in Table 2).

#### 3.1.3. Clinical Chemistry

A lower ALT when compared with d0 was found in the 20-mg/kg dose dogs on d14, the 40-mg/kg dose dogs on d28, and the 80-mg/kg dose dogs on d14 and d28. A lower AST when compared with d0 was found in the 20-mg/kg dose dogs on d14, the 40-mg/kg dose dogs on d14 and d28, and the 80-mg/kg dose dogs on d28. A lower ALP when compared with d0 was found in the 20-mg/kg dose dogs on d14 and d28, the 40-mg/kg dose dogs on d14, and the 80-mg/kg dose dogs on d28. A lower CPK level was found when compared with d0 in the 20- and 80-mg/kg dose dogs on d28 and the 40-mg/kg dose dogs on d14. A lower LDH when compared with d0 was found in the 20-mg/kg dose dogs on d14 and d28 and the 80-mg/kg dose dogs on d28 and d42. All the parameters mentioned above recovered after a 14-day drug-free period except for LDH. The other parameters fluctuated within the normal range. AST, ALP, CPK, TBIL, BU, TCH, TG, and CREA levels of animal ♀6 and ♀28, which were sacrificed on d8 or died on d16, were elevated when compared with the control group (only data with statistical significance are listed in Table 3).

#### 3.1.4. Urinalysis

There were no dose-related changes in the dogs’ urinalysis in the subchronic toxicity study.

#### 3.1.5. ECG Parameters

There were no dose-related changes in the dogs’ ECG examination in the subchronic toxicity study.

#### 3.1.6. Bone Marrow Examination

**Figure 2:**
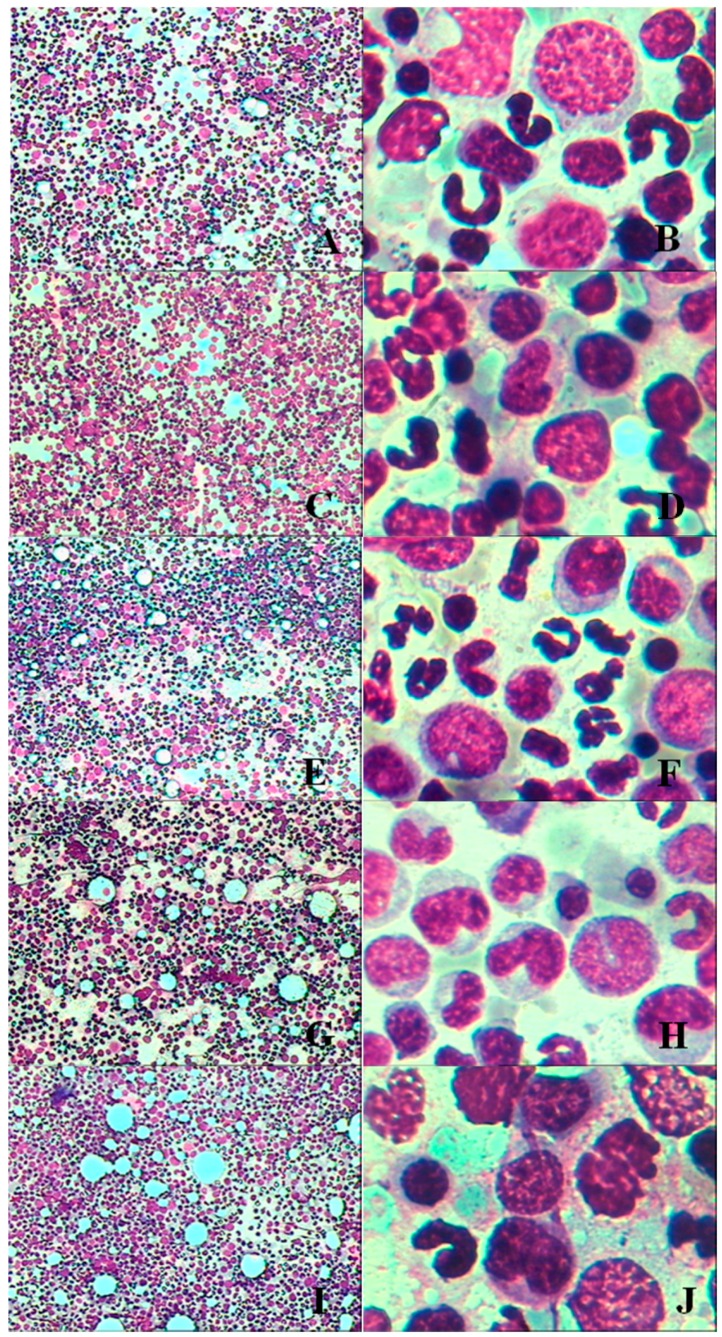
Bone marrow of dogs after treatments of HZ1006 (**A**–**J**) (**A**,**C**,**E**,**G**,**I**: original magnification × 100; **B**,**D**,**F**,**H**,**J**: original magnification × 1000). (**A**) photomicrograph of bone marrow from ♂29 (control group) on d28, original magnification × 100; (**B**) bone marrow from ♂29 (control group) on d28, original magnification × 1000; (**C**) bone marrow from ♀4 (5-mg/kg dose group) on d28, original magnification × 100; (**D**) bone marrow from ♀4 (5-mg/kg dose group) on d28, original magnification × 1000; (**E**) bone marrow from ♀22 (20-mg/kg dose group) on d28, original magnification × 100; (**F**) bone marrow from ♀22 (20-mg/kg dose group) on d28, original magnification × 1000; (**G**) bone marrow from ♂11 (40-mg/kg dose group) on d28, original magnification × 100; (**H**) bone marrow from ♂11 (40-mg/kg dose group) on d28, original magnification × 1000; (**I**) bone marrow from ♀4 (80-mg/kg dose group) on d28, original magnification × 100; (**J**) bone marrow from ♀29 (80-mg/kg dose group) on d28, original magnification × 1000. Note: Vacuolar degeneration that varied in amount and size was observed in the 20-, 40- and 80-mg/kg dose groups, and especially the 40- and 80-mg/kg dose groups. The numbers of nucleated red blood cells decreased to some extent, and bare nuclei and necrotic cells were observed in the bone marrow smears. Images captured from sections prepared from dogs euthanized at d28.

The main macroscopic findings in the dogs after the administration study included vacuolar degeneration that varied in amount and size that was observed in the 20-, 40- and 80-mg/kg dose groups, and especially in the 40- and 80-mg/kg dose groups. The main findings by oil immersion lens included decreased numbers of nucleated red blood cells and increased granulocyte counts. Additionally, bare nuclei and necrotic cells were observed in the bone marrow smears, which indicated that the test material had harmed or inhibited erythrocytes. Treatment-related bone marrow findings for the 28-days dogs study are shown in Figure 2.

#### 3.1.7. Necropsy

##### Gross Pathology and Organ Weights

The main macroscopic findings after administration for dogs included the following: pulmonary nodular protrusion and uneven and grainy shrinkage of the spleen in ♀6 dog at moribundity on d8 belonging to the 80-mg/kg dose group. Color changes of the lung, such as dark red or yellow white, were observed in ♂5 dog (lower lobe of right lung, belonging to the 80-mg/kg dose group, dark red), ♂23 dog (multifocal, belonging to the 40-mg/kg dose group, dark red) and ♂24 dog (middle lobe in right lung, belonging to the 40-mg/kg dose group, yellow white). Punctate protuberant lesions in the left lower lung lobe in ♂5 dog were also found. Two hard, grey red and soybean-like mediastinal lymph nodes as well as orchiatrophy were observed in ♂23 dog. These lesions mentioned above were found on d28. No other dose-related effects on organs or tissues were found. The absolute and relative weights of the lungs were elevated in ♂23 and ♂5 dogs belonging to the 40-mg/kg and 80-mg/kg dose groups, respectively, when compared with the control group on d28. The absolute and relative weights of the testes were reduced in the dogs of the 40-mg/kg and 80-mg/kg dose groups when compared with the control group (only data with statistical significance are listed in Table 4). Most of the organs were recovered after the drug-free period. There were no obvious changes observed in the weights of the other organs.

##### Histopathological Findings

*Lung*: The dogs numbered ♂5 and ♀6 (belonging to the 80-mg/kg dose group) and ♂23, ♀24 (belonging to the 40-mg/kg dose group) showed focal inflammation of the lungs, and their bronchiole walls became thicker than before and were obviously full of pyocytes. The alveoli surrounding the nidi disappeared and formed a confluent. Inside the pathological region, some structures of the bronchial wall were destroyed, the cilia fell off and large confluent bronchopneumonia pathological changes were found. Among these animals, the ♀24 dog was in relatively better condition than the other dogs with regard to pulmonary infection, as the purulent inflammation was mainly confined to the infected bronchial wall and the surrounding muscular alveolar septum. Microscopic examination revealed seriously enlarged and congested lymph nodes from ♂23 and ♂5 animals. No HZ1006 related histopathological findings were found in other dogs after 28 days of administration and 14 days of drug-free period.

*Bone marrow of sternum*: When compared with the control group, dose dependent increases in fat vacuoles, reduced red bone marrow proportions, and elevated yellow bone marrow ratios were observed in dogs belonging to the 20-, 40-, and 80-mg/kg dose groups, and each of these measurements recovered after the 14-day drug-free period.

##### *Male reproductive organs (testis, epididymis and* *prostate)*

- *Testis*: The numbers of seminiferous tubules decreased in dogs belonging to the 80- and 40-mg/kg dose groups, while the tubule lumen decreased and had reduced numbers and types of spermatogenic cells when compared with the control group. Only spermatogonia and some primary and secondary spermatocytes were observed, and there were no mature sperm compared with the control group.
- *Epididymis*: When compared with the control group, there was a notable lack of mature sperm cells and secretions in the epididymal lumen, as well as a decrease in epididymis lumen size, an irregular shape of the epididymal lumen and an increase in the connective tissue in the interstitium observed in dogs in the 80- and 40-mg/kg dose groups.
- *Prostate*: When compared with the control group, a reduction in the numbers and size, a thinner epithelium, narrower glandular cavity and increase in the connective tissue in the interstitium were observed in the prostatic acini of dogs in the 80- and 40-mg/kg dose groups.

**Figure 3:**
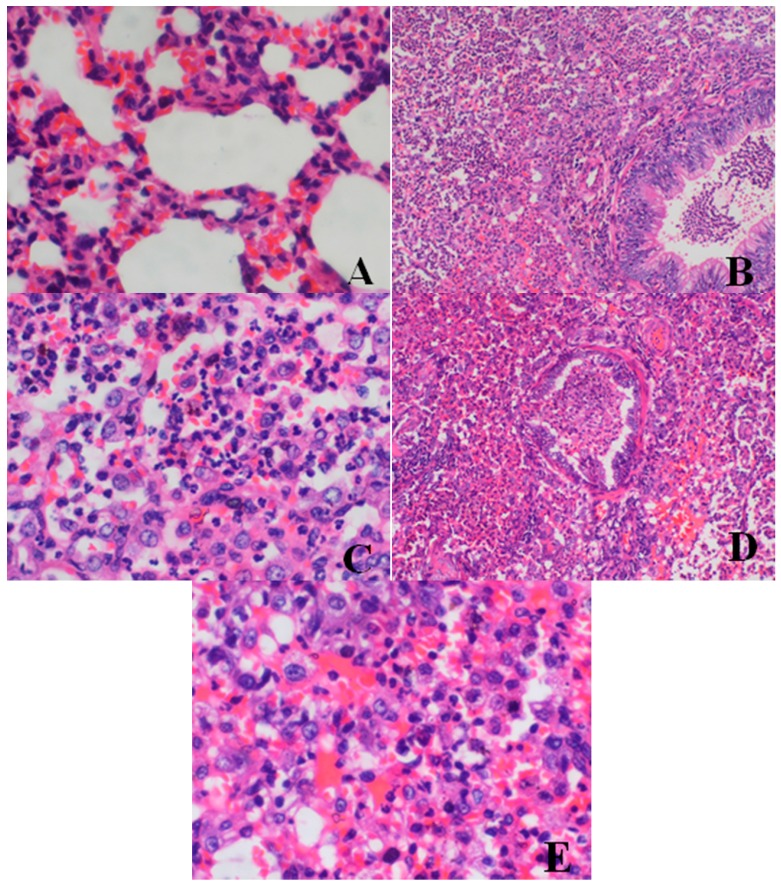
Hematoxylin and eosin-stained histologic sections of dogs’ lungs (**A**–**E**). (**A**) Histopathological photomicrograph of lung from ♀26 belonging to the control group at d28 (original magnification × 400): normal structure; (**B**) lung from ♂5 belonging to the 80-mg/kg dose group at d28 (original magnification × 100); (**C**) lung from ♂5 belonging to the 80-mg/kg dose group at d28 (original magnification × 400); (**D**) lung from ♂23 belonging to the 40-mg/kg dose group at d28 (original magnification × 100); (**E**) lung from ♂23 belonging to the 40-mg/kg dose group at d28 (original magnification × 400). Note the alveoli surrounding the nidi disappeared and formed a confluent lesion. Inside the pathological area, some structure of the bronchial wall was destroyed, cilia fell off and large confluent bronchopneumonia pathological changes occurred. Images captured from sections prepared from the necropsy of dogs euthanized at d28.

**Figure 4:**
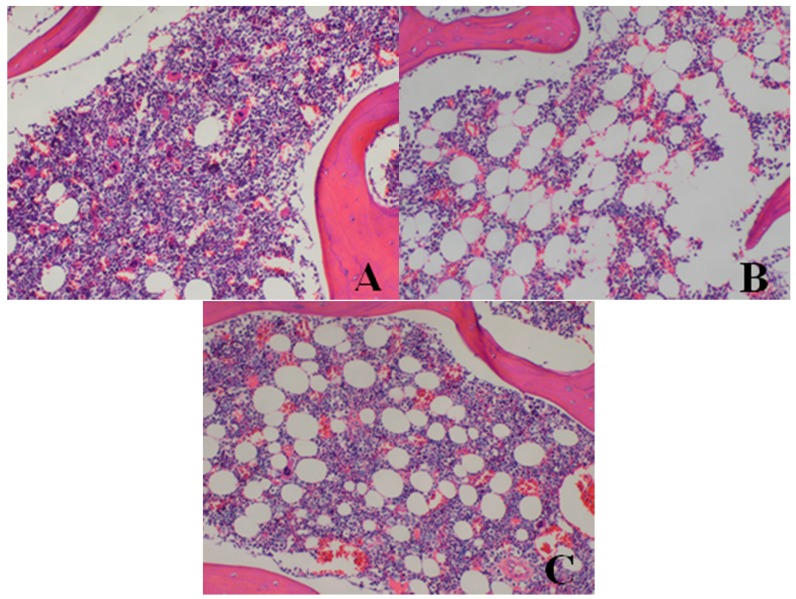
Hematoxylin and eosin-stained histologic sections of dogs’ bone marrow (**A**–**C**) (original magnification × 100). (**A**) Histopathological photomicrograph of bone marrow from ♀26 belonging to the control group at d28: normal; (**B**) Bone marrow from ♂5 belonging to the 80-mg/kg dose group at d28; (**C**) Bone marrow from ♂23 belonging to the 40-mg/kg dose group at d28. Note the increased fat vacuoles, decreased red bone marrow proportion, and increased yellow bone marrow ratio in the bone marrow. Images captured from sections prepared from the necropsy of dogs euthanized at d28.

**Figure 5:**
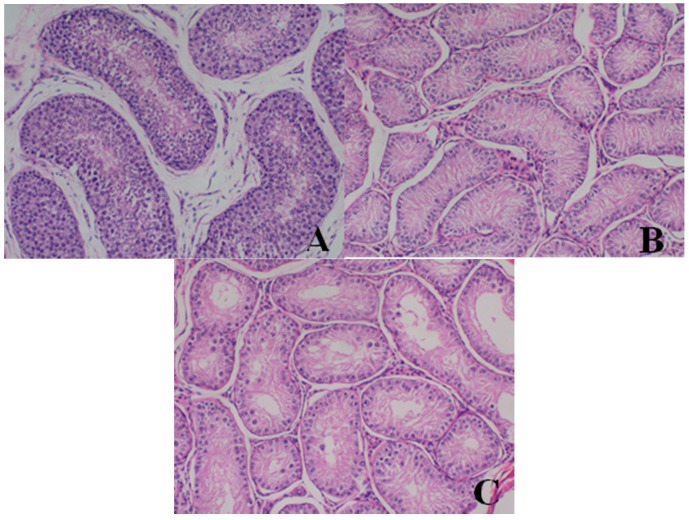
Hematoxylin and eosin-stained histologic sections of dogs’ testes (**A**–**C**) (original magnification × 100). (**A**) Histopathological photomicrograph of testis from ♂29 belong to the control group at d28: normal; (**B**) testis from ♂5 belonging to the 80-mg/kg dose group at d28; (**C**) testis from ♂23 belonging to the 40-mg/kg dose group at d28. Note that the tubule lumen decreased, and the numbers and types of spermatogenic cells were reduced. Images captured from sections prepared from the necropsy of dogs euthanized at d28.

**Figure 6:**
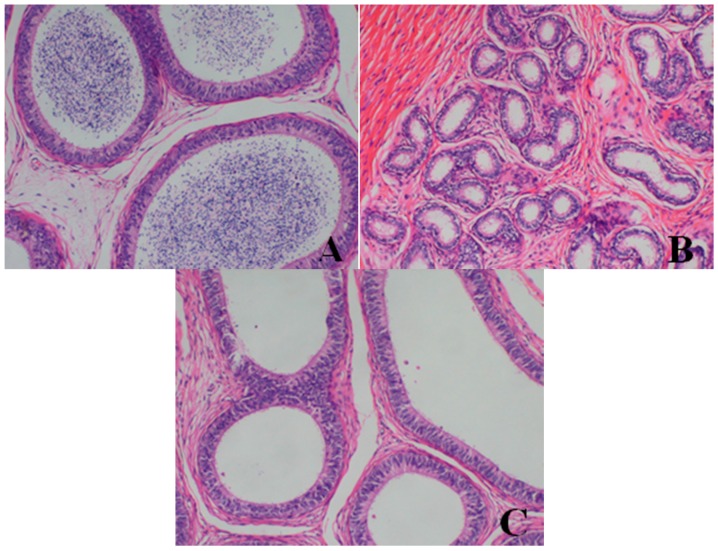
Hematoxylin and eosin-stained histologic sections of dogs’ epididymis (**A**–**C**) (original magnification × 100). (**A**) Histopathological photomicrograph of epididymis from ♂29 belonging to the control group at d28: normal; (**B**) Epididymis from ♂5 belonging to the 80-mg/kg dose group at d28: lack of mature sperm and secretions in the epididymal lumen, as well as a decrease in epididymal lumen size and the irregular shape of epididymal lumen; (**C**) Epididymis from ♂23 belonging to the 40-mg/kg dose group at d28: lack of mature sperm and secretions in the epididymal lumen. Images captured from sections prepared from the necropsy of dogs euthanized at d28.

**Figure 7:**
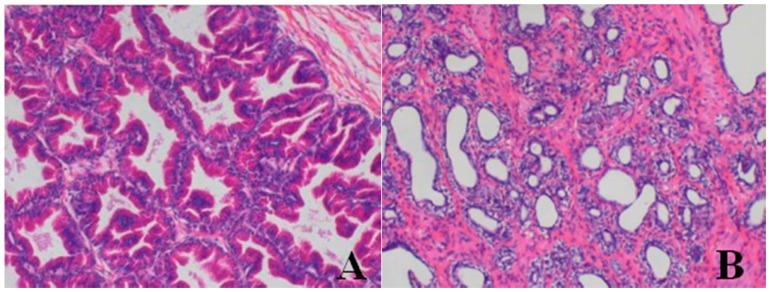

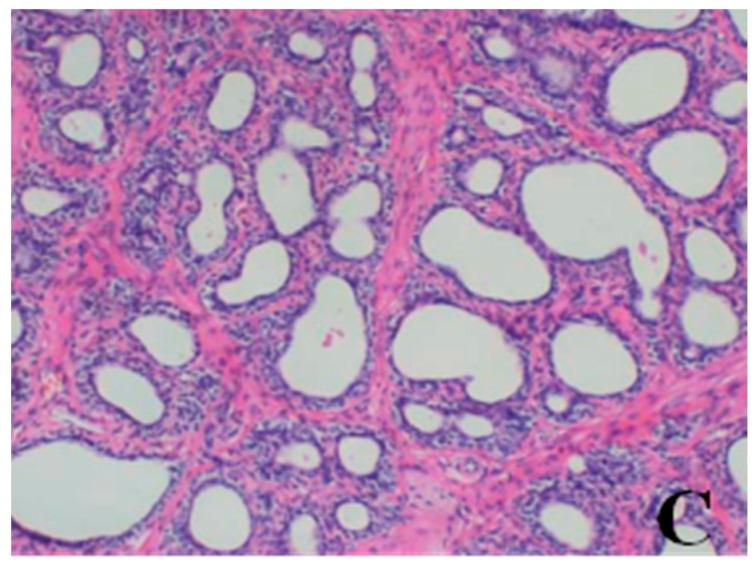
Hematoxylin and eosin-stained histologic sections of dogs’ prostates (**A**–**C**) (original magnification × 100). (**A**) Histopathological photomicrograph of the prostate from ♂29 belonging to the control group at d28: normal; (**B**) Prostate from ♂5 belonging to the 80-mg/kg dose group at d28; (**C**) Prostate from ♂23 belonging to the 40-mg/kg dose group at d28. Note the gland lumen narrowed, the thinner glandular epithelium, and the fewer acinar folds. Images captured from sections prepared from the necropsy of dogs euthanized at d28.

After a 14-day drug-free period, the situation in the testes, epididymis and prostate improved to some extent but did not recover completely. There were no obvious changes in the other groups. Dose-related histopathological findings for the 28-day dog study are shown in Figure 3, Figure 4, Figure 5, Figure 6 and Figure 7 and Table 5.

#### 3.1.8. Toxicokinetics

Pharmacokinetic parameters such as plasma AUC_0–∞_, C_max_ and T_max_, and T**_1/2_** were used to indicate the systemic exposure of the animals in our study. No significant differences were found in systemic exposure between male and female dogs during the 28-day study for the 5-, 20-, 40- and 80-mg/kg dose groups. Considerable inter-individual differences were observed in the plasma levels of HZ1006 in dogs. The plasma level of HZ1006 on the 1st, 14th and 28th day indicated that there was a dose-dependent and linear elevation with maximal plasma HZ1006 levels at 1–5 h after dosage. After 28 days of dosage of HZ1006, the accumulation coefficients R of the 5-, 20-, 40-, and 80-mg/kg dose groups were 1.3 ± 1.2, 3.5 ± 1.9, 0.7 ± 0.3 and 1.2 ± 0.9, respectively, when compared with d1, which indicated that there was some accumulation in dogs, although it was not significant in the 5–80 mg/kg dose range (data shown in Table 6).

### 3.2. Subchronic Toxicology Study on Rats

#### 3.2.1. Clinical Observation

**Figure 8:**
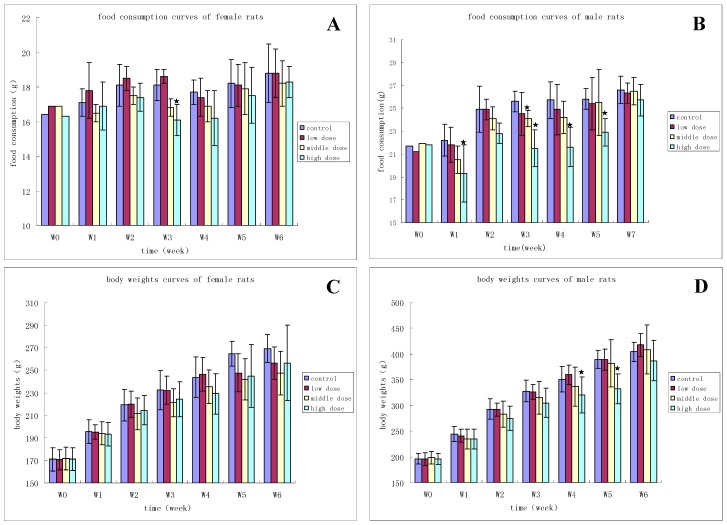
(**A**) Food consumption curves for female rats given HZ1006 for 28 days followed by a 14-day recovery; (**B**) Food consumption curves for male rats given HZ1006 for 28 days and followed by a 14-day recovery; (**C**) Body weight curves for female rats given HZ1006 for 28 days and followed by a 14-day recovery; (**D**) Body weight curves for male rats given HZ1006 for 28 days and followed by a 14-day recovery. ★ *p* < 0.05, compared with control group.

All rats survived until the scheduled necropsies. During the study period, there were no obvious adverse reactions in clinical symptoms. In week 3, female rats in the 60- and 120-mg/kg dose groups experienced reduced food consumption when compared with the control group. Male rats in the 120-mg/kg dose group in weeks 1, 3, 4, 5, and male rats in the 60-mg/kg dose in week 3 experienced the same reduction in food consumption. Body weights were significantly lower in weeks 4 and 5 for the male animals in the 120-mg/kg dose group when compared with the control group (reduced 8% and 15%, respectively). This phenomenon of body weight reduction improved during the other period of administration and the drug-free period. Obvious changes in the rats’ body weights and food consumption are shown in Figure 8.

#### 3.2.2. Hematology

There was a significant elevation of Ret in male rats in the 120-mg/kg dose group on d28 but not on d42 when compared with the control group. WBC and FIB were reduced on d42 in female animals in the 20- and 60-mg/kg dose groups, but not on d28 when compared with the control group. A significant reduction of APTT was found in female animals in the 20-mg/kg dose group, but all these values fluctuated within the normal ranges. A tendency toward an increase in NEUT % and a decrease in LYMPH % was found in female rats in all 3 dose groups on d28 and was only partially dose dependent (only results mentioned above are listed in Table 7). The changes of other hematological indexes were within normal ranges.

#### 3.2.3. Clinical Chemistry

The ALB in female rats belonging to the 60-mg/kg dose group was significantly reduced on d28, while AST and LDH decreased on d42 in male animals in the 60-mg/kg dose group when compared with the dose-free group. However, the changes of the three parameters above fluctuated within the normal ranges. The other values had no obvious changes induced by HZ1006.

#### 3.2.4. Urinalysis

There were no dose-related changes in urinalysis in the rats’ subchronic toxicity study.

#### 3.2.5. Bone Marrow Examination

There were no dose-related changes in the bone marrow examination in the rats’ subchronic toxicity study.

#### 3.2.6. Necropsy

##### Gross Pathology and Organ Weights

No obvious changes were observed in the rats’ organs or tissues by macroscopic examination. Higher absolute weights of the adrenal glands and lower relative weights of the brain in female rats in the 20-mg/kg dose group were observed compared with the dose-free group on d28. Higher absolute weights of the testes were observed in male rats in the 20-, 60- and 120-mg/kg dose groups when compared with the 0-mg/kg dose group on d42, but no changes were found in relative weights. The data mentioned above fluctuated within the normal ranges. There were lower relative weights of the kidneys in male rats belonging to the 20-, 60- and 120-mg/kg dose groups when compared with the control group on d28, and no obvious changes in the 20- and 60-mg/kg dose groups on d42. However, the relative weights of the male rats’ kidneys in the 120-mg/kg dose group had not recovered (only results with statistical significance are list in Table 8). Other values had no obvious changes induced by HZ1006.

##### Histopathological Findings

No dose-related histopathological findings were observed in the subchronic toxicity study.

#### 3.2.7. Toxicokinetics

Pharmacokinetic parameters, such as plasma AUC_0–∞_, C_max_ and T_max_, and T**_1/2,_** were used to indicate systemic exposure of animals in our study. No significant differences were found in the systemic exposure between male and female rats during the 28 day study for the 20-, 60- and 120-mg/kg dose groups. Considerable inter-individual differences were observed in the plasma level of HZ1006 of the rats. The plasma level of HZ1006 on the 1st and 28th day indicated that there was a dose-dependent and linear increase, with maximum plasma HZ1006 levels 0.17–8.0 h after dosage. After 28 days of dosage of HZ1006, accumulation coefficients R of 20-, 60- and 120-mg/kg dose groups were 16.8 ± 21.6, 3.2 ± 1.2, and 6.3 ± 2.8, respectively, when compared with d1, which indicated there was some accumulation in the rats after 28 days of repeated administration when in the 20–120-mg/kg dose range (data shown in Table 9).

## 4. Discussion

The determination of HZ1006 dosage was mainly according to our preliminary experiments. The selection of the low dose for the repeated dose toxicity tests was based on the no toxic effect dose, whereas the high dose was definite when considering toxicity. Therefore, based on our preliminary experiment results, estimated clinical dose (2–5-mg/kg/day) and the pharmacodynamic dose in vitro test of HZ1006, doses of 0, 5, 20, 40 and 80 mg/kg/day for dogs and 0, 20, 60, 120 mg/kg/day for rats were selected.

The daily administration of HZ1006 by gavage at dosage levels of 0, 20, 60 and 120 mg/kg/day to rats in the 28-day study was tolerated well, and no other side effects were observed except for loss of weight and decreased food consumption in animals belonging to the 60- and 120-mg/kg/day groups. After repeated dosage of HZ1006, some intrinsically associated symptoms, including inappetence, repeated vomiting, and diarrhea accompanied by a slow increase in body weight and an increase in relative brain weight in dogs, were observed. The symptoms mentioned above suggested that HZ1006 might impact the digestive system after repeated dosage. The hypothesis that the digestive system might be one of the toxic targets of HZ1006 was also proven by the unplanned death of dogs in the 80- and 40-mg/kg dose groups that exhibited decreased body weights and rectal temperature and elevated levels of AST, ALP, TBIL, Tch, and TG at moribundity after several days of administration. Decreases in K^+^, EOS, ALT, AST, LDH and CPK as well as swelling of the limbs and erubescence was also observed in some dogs belonging to the 20-, 40- and 80-mg/kg/day groups. The side effects mentioned above might be caused by hepatic toxicity and increased local HZ1006 levels. The uncomfortable symptoms, however, such as squatting, prostration, reduced activity, salivation and tremors induced by the stimulation of gastrointestinal tract or nervous system by the test sample, require further research and clinical trials. Much of the adverse reactions conformed to the pharmacological effects of HZ1006 and other HDAC inhibitors such as Vorinostat. The most prominent drug-related side effects included reduced body weight, food consumption, diarrhea, and hematologic changes in both female and male animals following dosage of Vorinostat for 26 weeks at doses of more than 50 mg/kg/day [7]. Although many of these effects were consistent with other cytotoxic drugs (inappetence, weight loss, and hematologic effects), the gastrointestinal system findings in the dogs were deemed to be HZ1006-related adverse findings, though the mechanism of action requires additional research.

The end points indicating toxicologic hematologic effects on dogs included reductions of RBC, HCB, HCT and RET, elevation of APTT and FIB as well as the changes in Plat and PT of the unscheduled dead animals. The effects on erythrocytes in the bone marrow were consistent with histopathologic findings. Erythroid hypoplasia was evident in the bone marrow smears and bone marrow histologic sections after 28 days of dosing. The effect on NEUT % and LYMPH % of female rats in the high dose group was more obvious compared with the control group. The side effects indicated toxicity of the hematologic system, which may be the result of the active role of HZ1006 in inducing cell growth arrest, differentiation or apoptosis by interfering with the cell cycle. Results showed that the hematopoietic system was the predominantly affected organ by HZ1006 in our studies, especially at high doses, which conformed to the current understanding of the mechanism of action of HDACIs.

Obvious elevation of lung weight and multifocal bronchopneumonia were found after 28 days of repeated administration in dogs belonging to 40- and 80-mg/kg dose groups, and these findings indicated that the respiratory system might be one of HZ1006’s toxic targets. The fact that HZ1006 might influence the respiratory system is consistent with the symptom of hyperpnea of some dogs after continuous dosing. Increasing evidence suggests that some HDACIs might affect the respiratory system, such as vorinostat in phase 2 trials, which included pulmonary embolism and thrombosis [8]. Therefore, the toxicity on the respiratory system induced by HZ1006 in our dose-repeated studies might be because of deacetylation of both histone and non-histone proteins by this drug candidate. However, when combining the fact that the drug had inhibitory effects on the bone marrow system, it is also indicated that the cause of the large fusion of acute bronchial pneumonia of the dogs may be a secondary infection induced by the test compound. When considering the fact that the dogs repeatedly vomited, the possibility of vomit causing aspiration pneumonia should not be excluded.

The absolute and relative weights of the testes of male dogs in the 40- and 80-mg/kg dose groups decreased, and the male reproductive organ (including the testes, epididymis, and prostate) lesions included inhibited spermatogenic function that were common in these dogs, which showed that the male reproductive system may be one of the toxic targets of HZ1006. Although we still have no idea about the real mechanism of HZ1006’s toxicity on the male reproductive system, some research has shown that this toxicity may be related to the inhibition of the androgen receptor (AR). In AR regulation, some specific HDAC enzymes, including HDAC1, 3 and 6, might play very important roles. Welsbie et al. have shown that the expression of AR-regulated genes can be suppressed and mirror the effect of HDACI therapy when the HDAC1 (and HDAC3) gene was knocked down [9]. HSP90, one of the ATPase-driven molecular chaperones, has very important effects on oncogenic tyrosine and serine–threonine kinases, such as estrogen receptors and AR, which belong to steroid hormone receptors and can sustain molecular stability, conformation and function [10]. Fiskus et al. confirmed that HSP90 chaperone activity is regulated by HDAC6, and in HDAC6-deficient cells, HSP90-dependent post-translational maturation of AR, one of the nuclear steroid receptors, might be disrupted [11]. Hyperacetylation of HSP90 can be induced by romidepsin, one of the HDACIs, and then the complex between HSP90 and its client proteins can be destroyed, which could inhibit their synthesis and function [12]. LAQ824 (one of the HDACIs) can reduce wild-type and mutated AR through HSP90 inhibition when treated in androgen-dependent and -independent prostate cancer cell lines, and in LNCaP cells, the drug also affected androgen-induced prostate-specific antigen (PSA) production, blocked cell proliferation and resulted in apoptosis [13]. The toxic effect of HZ1006 on reproductive systems may be the extension of pharmacological action that can explain the mechanism of side effects of other similar drugs on prostate cancer and castration-resistant prostate cancer (CRPC); in other words, HZ1006 may be particularly important in helping to eliminate the broad-spectrum inhibitor constitutional toxic effects via selecting HDACIs targets.

In addition to the safety profiles mentioned above, mechanism-based cardiac toxicities were also a substantial issue raised by recent studies about cases of QT interval prolongation among HDACIs [2], although the results from our studies showed that HZ1006 had no obvious adverse reaction on the cardiovascular system of animals after long-term dosage. Considering the differences in adverse effects of cardio-toxicity between HZ1006 and other HDAC inhibitors, HZ1006 appeared to be less toxic than other HDACIs in cardiovascular toxicity, although the special safety pharmacology cardiovascular evaluations of HZ1006 are not yet available. To avoid unnecessary risks, precautionary measures, such as selection of patients without a significant cardiac history, intensive monitoring of ECGs, heart rate, QTc and electrolyte levels, as well as opting for intermittent instead of daily dosing schedules in patients who take HZ1006 clinically, are necessary and very important.

Pharmacokinetic parameters, such as plasma AUC_0–∞_, C_max_ and T**_max_**, T**_1/2_** used to indicate rats and dogs’ systemic exposure to HZ1006, illustrated that plasma HZ1006 levels were proportional to the dose, and no saturation was observed. After 28 days of oral administration of HZ1006, accumulation coefficients R of the 20-, 60-, 120- and 5-, 20-, 40-, 80-mg/kg dose groups in rats and dogs, respectively, were higher than 1 when compared with d1, which indicated there was some accumulation in rats and dogs after 28 days of repeated administration when in 20–120-mg/kg and 5–80-mg/kg dose range, respectively.

Despite structural diversity, the HDACIs family shares a similar mode of action and generates a uniform class toxicity profile, which includes gastrointestinal effects, dose-related transient cytopenias and male reproductive organ lesions that are rarely life threatening and generally well manageable either by dosing delay or dose reduction. However, because differences exist between HZ1006 and other HDACIs, and the reasons remain unknown, we should try our best to understand the structure-activity relationship and HZ1006’s molecular mechanisms.

## 5. Conclusions

According to our research, we conclude the following: the NOAEL of HZ1006 by repeated oral dosage for dogs and rats was 5 mg/kg and 60 mg/kg, respectively, and the minimum toxic dose was 20 and 120 mg/kg, respectively. HZ1006’s toxic targets might be the digestive tract, the male reproductive tract, the respiratory tract and the hematological systems, and most lesions induced by HZ1006 are reversible. There are both similarities and differences in toxic characteristics between HZ1006 and other HDACIs. Although the reasons for the differences remain unknown, it is expected that HZ1006 could be a feasible and safe substitution for other existing HDACIs if it becomes certified as effective and safe in upcoming clinical trials.

## Figures and Tables

**Table 1:** Selected clinical signs induced by HZ1006 in dogs following repeated dose administration during the study of subchronic toxicity (only data regarding the frequencies of vomiting and diarrhea during test time are listed).

Animal Number	Frequencies of Vomiting (Days)	Frequencies of Diarrhea (Days)	Animal Number	Frequencies of Vomiting (Days)	Frequencies of Diarrhea (Days)
Control					
♂9	0	0	♀14	0	0
♂21	0	0	♀26	0	0
♂29	0	0	♀30	0	0
♂33	0	0	♀36	0	0
5 mg/kg					
♂7	0	0	♀4	0	0
♂31	0	0	♀34	0	0
♂25	0	0	♀38	0	0
♂35	0	0	♀42	0	0
20 mg/kg					
♂15	2	0	♀12	1	0
♂19	0	0	♀16	6	0
♂27	2	0	♀22	1	0
♂37	1	0	♀32	4	0
40 mg/kg					
♂1	6	1	♀10	1	2
♂11	9	8	♀20	6	0
♂23	9	1	♀24	3	2
♂39	5	3	♀28	4	3
80 mg/kg					
♂3	9	3	♀2	5	8
♂5	2	11	♀6	4	0
♂13	12	5	♀8	3	2
♂17	4	2	♀18	6	9

**Table 2:** Selected hematological parameters induced by HZ1006 in Beagle dogs following repeated dose administration during the study of subchronic toxicity (only significant results are listed).

Items	Days	Control	5 mg/kg	20 mg/kg	40 mg/kg	80 mg/kg	♀6 ^a^	♀28 ^b^
RBC (×10^12^/L)	d0	6.29 ± 0.68	6.31 ± 0.45	6.20 ± 0.58	6.01 ± 0.31	5.92 ± 0.42		
	d14	6.51 ± 0.76	6.23 ± 0.46	5.57 ± 0.30 ^##,^*	5.6 ± 0.36 ^##,^*	5.09 ± 0.42 ^##^^,^*		
	d28	6.50 ± 0.54	6.22 ± 0.43	5.57 ± 0.16 ^##,^*	5.00 ± 0.47 ^##,^*	4.17 ± 0.33 ^##,^**		
	d42	6.27 ± 0.36	6.21 ± 0.36	5.96 ± 0.13	5.96 ± 0.06	5.71 ± 0.50		
HCT (%)	d0	42.4 ± 4.4	43.0 ± 2.6	42.2 ± 3.9	41.7 ± 2.0	40.3 ± 3.0		
	d14	43.2 ± 4.3	41.8 ± 2.7	36.9 ± 2.2 ^##,^*	35.5 ± 2.6 ^##,^**	33.4 ± 2.6 ^##,^*		
	d28	42.7 ± 3.0	41.6 ± 2.4	36.6 ± 1.0 ^##,^**	32.8 ± 2.8 ^##,^**	26.9 ± 1.7 ^##,^**		
	d42	41.8 ± 2.3	41.0 ± 1.8	39.4 ± 1.7	39.7 ± 0.1	37.5 ± 3.1		
HGB (g/L)	d0	142 ± 14	139 ± 8	142 ± 13	139 ± 6	134 ± 10		
	d14	139 ± 14	138 ± 9	118 ± 7 ^##,^**	114 ± 8 ^##,^**	107 ± 8 ^##,^**		
	d28	137 ± 9	135 ± 8	118 ± 3 ^##,^**	105 ± 9 ^##,^**	86 ± 5 ^##,^**		
	d42	136 ± 7	134 ± 7	128 ± 8	124 ± 1	126 ± 4		
RET (%)	d0	0.87 ± 0.34	0.77 ± 0.38	0.96 ± 0.24	0.86 ± 0.17	0.92 ± 0.27	1.37	0.75
	d14	0.63 ± 0.29	0.62 ± 0.29	0.53 ± 0.23 *	0.13 ± 0.11 ^##,^**	0.08 ± 0.12 ^##,^**	0.14	0.04
	d28	0.48 ± 0.21	0.49 ± 0.23	0.54 ± 0.19 **	0.51 ± 0.22*	0.21 ± 0.16 ^#,^**		
	d42	0.65 ± 0.25	0.61 ± 0.22	0.83 ± 0.23	0.89 ± 0.65	0.88 ± 0.27		
MON (%)	d0	4.8 ± 1.0	4.4 ± 1.6	4.7 ± 1.3	6.0 ± 1.8	5.0 ± 0.8		
	d14	4.5 ± 1.3	4.2 ± 1.0	8.0 ± 2.3 **	9.2 ± 5.3	19.7 ± 19.7 ^#^		
	d28	4.7 ± 1.0	4.4 ± 1.4	9.0 ± 1.4 ^#,^**	11.6 ± 4.5 ^##^	12.5 ± 2.9 ^##,^**		
	d42	4.7 ± 1.0	4.0 ± 1.2	5.8 ± 1.0	6.4 ± 3.5	5.0 ± 1.9		
APTT (s)	d0	12.7 ± 0.8	13.6 ± 0.6	12.4 ± 0.3	12.8 ± 0.5	13.4 ± 0.6	13.9	12.4
	d14	13.0 ± 0.9	13.2 ± 0.6	12.5 ± 0.5	16.8 ± 0.7 ^##,^**	16.2 ± 3.0 ^##^	48.7	18.5
	d28	12.1 ± 0.9	13.1 ± 0.5	12.0 ± 0.7	12.8 ± 0.8	12.5 ± 0.5 **		
	d42	13.8 ± 1.0	13.4 ± 0.4	12.8 ± 0.3	12.9 ± 0.8	12.7 ± 0.9		
FIB (g/L)	d0	3.86 ± 0.86	2.25 ± 0.37	4.07 ± 0.94	4.84 ± 0.89	3.80 ± 0.84		
	d14	3.93 ± 1.26	2.53 ± 0.46	4.30 ± 1.17	7.19 ± 1.58 ^##^	7.06 ± 2.14 ^##,^**		
	d28	3.18 ± 0.58	2.56 ± 0.40	4.48 ± 1.68	4.93 ± 1.22	6.69 ± 0.62 ^##,^**		
	d42	2.96 ± 0.70	2.89 ± 0.60	4.26 ± 0.49	4.22 ± 1.25	3.72 ± 2.00		

^a^ One female dog ♀6 belonging to the 80-mg/kg dose group demonstrated moribundity on d8 and was then euthanized to obtain the data shown in the d14 line, although these data was not included in the mean values ± SD; ^b^ One female dog ♀28 in the 40-mg/kg dose group died on d16, and its data on d14 was not included in the mean values ± SD; RBC: Red blood cell count; HCT: Hematocrit; HGB: Hemoglobin concentration; RET: Reticulocytes; MON: Monocyte; APTT: Activated partial thromboplastin time; FIB: Fibrinogen; * *p* < 0.05, ** *p* < 0.01 compared with d0; ^#^ *p* < 0.05, ^##^ *p* < 0.01 compared with the control group; data represent the mean values ± SD. The data on the other female dog ♀8 that belonged to the 80-mg/kg dose group that died on d10 was not collected.

**Table 3:** Selected serum biochemistry parameters induced by HZ1006 in Beagle dogs following repeated dose administration during the study of subchronic toxicity (only significant results are listed).

Items	Days	Control	5 mg/kg	20 mg/kg	40 mg/kg	80 mg/kg	♀6 ^a^	♀28 ^b^
ALT (nmol/s/L)	d0	590 ± 167	559 ± 120	535 ± 92	526 ± 179	591 ± 143	487	428
	d14	563 ± 196	521 ± 116	425 ± 105 **	433 ± 304	312 ± 38 *	466	852
	d28	611 ± 155	593 ± 105	486 ± 90	347 ± 78 *	307 ± 51 **		
	d42	562 ± 165	558 ± 90	523 ± 148	514 ± 112	549 ± 181		
AST (nmol/s/L)	d0	436 ± 85	463 ± 106	456 ± 92	462 ± 57	493 ± 103	456	398
	d14	472 ± 72	442 ± 105	384 ± 56 *	313 ± 23 **	386 ± 104	1907	4924
	d28	419 ± 68	424 ± 109	408 ± 57	378 ± 71 *	310 ± 59 **		
	d42	470 ± 68	451 ± 79	412 ± 31	468 ± 80	460 ± 83		
LDH (μmol/s/L)	d0	3.02 ± 1.38	3.08 ± 1.36	2.85 ± 0.86	3.39 ± 1.55	2.71 ± 0.97	2.78	2.21
	d14	2.28 ± 0.37	2.27 ± 1.24	1.23 ± 0.45 **	1.29 ± 0.68	1.76 ± 1.85	2.91	2.06
	d28	2.59 ± 0.45	2.71 ± 1.12	1.54 ± 0.74 *	2.76 ± 2.49	0.97 ± 0.30 **		
	d42	2.25 ± 0.27	2.21 ± 1.32	2.28 ± 0.93	3.50 ± 2.49	3.20 ± 1.62 **		
CPK (μmol/s/L)	d0	2.98 ± 0.63	2.94 ± 1.40	2.98 ± 0.64	3.27 ± 0.76	3.24 ± 0.77	3.06	2.47
	d14	2.78 ± 0.27	2.63 ± 1.36	2.43 ± 0.48	2.36 ± 0.19 *	3.29 ± 1.86	57.57	6.39
	d28	2.58 ± 0.29	2.43 ± 0.19	2.38 ± 0.53 **	3.00 ± 0.94	1.73 ± 0.24 **		
	d42	2.56 ± 0.20	2.97 ± 0.18	2.17 ± 0.30	3.00 ± 1.32	2.86 ± 0.53		
ALP (μmol/s/L)	d0	1.55 ± 0.43	1.47 ± 0.30	1.48 ± 0.48	1.62 ± 0.40	1.62 ± 0.49	1.50	1.33
	d14	1.49 ± 0.28	1.45 ± 0.35	0.85 ± 0.25 **	1.03 ± 0.32 **	1.27 ± 1.15	3.86	3.16
	d28	1.52 ± 0.48	1.51 ± 0.29	0.98 ± 0.27 **	1.30 ± 0.24	1.01 ± 0.27 **		
	d42	1.27 ± 0.20	1.45 ± 0.37	1.00 ± 0.24	1.34 ± 0.34	0.99 ± 0.14		
TBIL (μmol/L)	d0	2.50 ± 0.30	2.34 ± 0.31	2.55 ± 0.28	2.24 ± 0.24	2.64 ± 0.41	2.95	2.18
	d14	2.17 ± 0.46	2.01 ± 0.41	1.85 ± 0.16	1.81 ± 0.24	2.74 ± 2.93	11.53	9.65
	d28	2.17 ± 0.36	2.08 ± 0.52	2.29 ± 0.31	2.04 ± 0.43	2.19 ± 0.20		
	d42	1.99 ± 0.39	2.01 ± 0.33	1.88 ± 0.19	1.88 ± 0.08	1.90 ± 0.23		
BU (mmol/L)	d0	4.29 ± 1.07	4.03 ± 0.52	4.35 ± 0.98	3.95 ± 0.48	4.38 ± 0.72	4.04	4.08
	d14	4.00 ± 0.75	4.10 ± 0.90	4.52 ± 1.05	4.52 ± 1.05	4.66 ± 0.93	15.20	4.89
	d28	3.63 ± 0.31	3.82 ± 0.51	5.37 ± 1.78	4.66 ± 0.96	5.25 ± 1.21		
	d42	4.74 ± 0.40	4.83 ± 0.20	4.05 ± 0.37	4.87 ± 1.56	4.93 ± 0.50		
TCH (mmol/L)	d0	4.45 ± 0.70	4.28 ± 0.36	4.31 ± 0.36	5.38 ± 1.95	4.03 ± 1.04	3.27	5.85
	d14	4.08 ± 0.78	3.98 ± 0.64	3.16 ± 0.34	4.47 ± 0.71	4.62 ± 2.51	11.04	10.73
	d28	4.48 ± 0.80	4.24 ± 0.56	3.95 ± 0.55	4.66 ± 1.85	4.16 ± 1.39		
	d42	4.12 ± 1.21	3.99 ± 0.41	3.81 ± 0.18	4.55 ± 1.10	3.52 ± 0.84	0.31	0.39
TG (mmol/L)	d0	0.43 ± 0.12	0.43 ± 0.11	0.42 ± 0.09	0.41 ± 0.10	0.38 ± 0.15	1.60	1.40
	d14	0.38 ± 0.15	0.39 ± 0.08	0.29 ± 0.08	0.41 ± 0.12	0.56 ± 0.28		
	d28	0.33 ± 0.08	0.35 ± 0.11	0.34 ± 0.05	0.43 ± 0.18	0.45 ± 0.11		
	d42	0.29 ± 0.05	0.31 ± 0.09	0.33 ± 0.12	0.45 ± 0.04	0.40 ± 0.14		
CREA (μmol/L)	d0	50.9 ± 7.9	51.5 ± 9.3	51.6 ± 7.5	49.2 ± 6.2	53.4 ± 5.2	52.8	47.9
	d14	50.3 ± 5.5	51.0 ± 9.9	58.6 ± 10.6	52.5 ± 6.1	58.2 ± 4.8	82.4	37.8
	d28	50.9 ± 5.2	51.4 ± 10.9	52.1 ± 10.3	52.6 ± 9.8	54.3 ± 8.9		
	d42	56.4 ± 5.7	55.6 ± 4.5	49.6 ± 0.6	58.8 ± 2.0	65.9 ± 11.3		

^a^ One female dog ♀6 belonging to the 80-mg/kg dose group demonstrated moribundity on d8 and was then euthanized to obtain the data shown in the d14 line, although these data was not included in the mean values ± SD; ^b^ One female dog ♀28 in the 40-mg/kg dose group died on d16, and its data on d14 was not included in the mean values ± SD; ALT: Alanine aminotransferase; AST: Aspartate aminotransferase; LDH: Lactate dehydrogenase; CPK: Creatine phosphokinase; ALP: Alkaline phosphatase; TBIL: Total bilirubin; BU: Blood urea; TCH: Total cholesterol; TG: Triglycerides; CREA: Creatinine; * *p* < 0.05, ** *p* < 0.01 compared with d0; compared with the control group; data represent the mean values ± SD. another female dog ♀8 from the 80-mg/kg dose group died on d10, and the data from this dog was not obtained.

**Table 4:** Selected absolute and relative organ weights induced by HZ1006 in dogs following repeated dose administration after 28 days of continuous treatment and 14 days of recovery (only significant results are listed).

Organ	Days	Control	5 mg/kg	20 mg/kg	40 mg/kg	80 mg/kg	♂23	♂5
Absolute weight								
Lung (g)	d28	69.9 ± 3.0	72.7 ± 8.2	71.4 ± 12.6	92.4 ± 15.4	95.8 ± 35.3	109.8	135.9
	d42	74.7 ± 7.1	±	74.0 ± 8.8	70.4 ± 5.0	82.9 ± 6.8		
Testis (g)	d28	13.625 ± 0.714	13.550 ± 0.494	13.953 ± 0.081	6.631 ± 2.855 **	5.660 ± 1.608 **		
	d42	12.820 ± 1.556	14.050 ± 0.212	12.179 ± 0.518	9.400 ± 0.063 **	6.017 ± 2.937 **		
Relative weight								
Lung	d28	6.93 ± 0.52	6.90 ± 0.90	6.96 ± 1.02	9.87 ± 2.14	9.87 ± 3.27	12.34	13.39
	d42	7.46 ± 0.43	±	7.37 ± 0.67	7.10 ± 1.13	8.49 ± 0.95		
Testis	d28	1.44 ± 0.02	1.23 ± 0.03	1.31 ± 0.26	0.69 ± 0.24 **	0.61 ± 0.23 **		
	d42	1.29 ± 0.08	1.45 ± 0.04	1.21 ± 0.01	0.78 ± 0.21 **	0.63 ± 0.31 **		

* *p* < 0.05, ** *p* < 0.01 compare with d0, compare with the control group, the data represent the mean values ± SD.

**Table 5:** Selected histopathological findings in dogs after 28 days of treatment with HZ1006.

Organ	Findings	Dose (mg/kg/day)				
		0	5	20	40	80
Lung	large confluent bronchopneumonia	0/4	0/4	0/4	2/4	2/4
Bone marrow of sternum	decrease in hematopoietic cell numbers	0/4	0/4	0/4	2/4	3/4
Testis	Reduction in numbers and types of spermatogenic cells	0/2	0/2	0/2	2/2	2/2
Epididymis	lack of mature sperm and secretions in the epididymal lumen as well as decrease of epididymal lumen size	0/2	0/2	0/2	2/2	2/2
Prostate	reduction in numbers and size, thinner epithelium, narrowing of the glandular cavity of prostatic acini	0/2	0/2	0/2	2/2	2/2

Dogs (4/sex/group) were treated orally with HZ1006 for 28 days, and one-half of the animals were sacrificed; the remaining half was sacrificed after 14 days of recovery. The data represent the number examined (bold) and incidence.

**Table 6:** Mean (± SD) toxicokinetic parameters of HZ1006 after oral administration in doses of 5, 20, 40 and 80 mg/kg/day to Beagle dogs.

Dose (mg/kg/day)	Duration of Administration	AUC_0–__∞_ (ng·h·mL^−1^)	C_max_ (ng·mL^−1^)	T_max_ (h)	t_1/2_ (h)
5 (*n* = 6)	single	193.1 ± 64.6	35.6 ± 15.1	3.67 ± 1.52	4.40 ± 0.61
	14 days	198.9 ± 69.6	30.1 ± 14.2	4.20 ± 2.01	4.75 ± 1.23
	28 days	616.1 ± 242.4	65.7 ± 42.0	4.33 ± 2.94	7.34 ± 1.02
20 (*n* = 6)	single	1191.2 ± 319.9	139.1 ± 73.4	3.50 ± 2.51	5.54 ± 1.53
	14 days	620.3 ± 248.7	68.1 ± 22.8	4.00 ± 2.19	5.21 ± 0.53
	28 days	388.9 ± 103.8	55.2 ± 21.8	3.17 ± 1.33	5.35 ± 0.73
40 (*n* = 6)	single	2500.4 ± 1490.8	456.0 ± 375.5	2.70 ± 2.70	6.93 ± 3.03
	14 days	1021.5 ± 976.6	199.0 ± 174.6	1.90 ± 1.34	7.34 ± 3.82
	28 days	927.9 ± 297.5	162.2 ± 101.6	2.20 ± 1.10	5.08 ± 1.04
80 (*n* = 6)	single	3874.9 ± 2632.8	365.4 ± 256.7	3.50 ± 2.51	6.74 ± 2.20
	14 days	1547.4 ± 758.0	173.5 ± 123.0	4.43 ± 3.53	7.23 ± 3.45
	28 days	1820.9 ± 570.0	180.2 ± 83.4	4.60 ± 3.29	7.56 ± 3.27

AUC_0–∞_: Area under curve; C_max_: Maximum concentrations; T_max_: Maximum time; t_1/2_: Half-time.

**Table 7:** Selected hematological parameters induced by HZ1006 in rats following repeated dose administration during the study of subchronic toxicity (only significant results are listed).

Items	Days	Control	20 mg/kg	60 mg/kg	120 mg/kg
Female					
WBC	d28	3.32 ± 0.54	3.17 ± 1.23	2.82 ± 0.60	3.95 ± 0.77
(×10^9^·L^−1^)	d42	3.78 ± 0.92	2.68 ± 0.63 *	2.53 ± 0.60 *	3.00 ± 0.43
FIB	d28	1.78 ± 0.11	1.87 ± 0.09	1.89 ± 0.06	2.01 ± 0.40
(g/L)	d42	1.89 ± 0.19	1.62 ± 0.17 *	1.62 ± 0.18 *	1.85 ± 0.12
APTT	d28	24.3 ± 1.7	24.3 ± 3.5	23.0 ± 2.4	22.6 ± 2.4
(s)	d42	26.2 ± 2.0	22.2 ± 3.0 **	25.2 ± 1.3	25.2 ± 1.6
Neu	d28	8.40 ± 0.80	8.70 ± 1.72	12.08 ± 4.77	19.28 ± 8.26
(%)	d42	12.42 ± 1.73	11.58 ± 4.04	12.06 ± 6.70	10.76 ± 1.81
Lym	d28	88.40 ± 0.77	88.38 ± 1.68	84.70 ± 5.45	77.22 ± 8.78
(%)	d42	84.30 ± 1.57	85.40 ± 3.85	85.36 ± 6.93	85.88 ± 1.85
Males					
RET	d28	1.61 ± 0.21	1.88 ± 0.32	1.43 ± 0.22	2.04 ± 0.31 *
(%)	d42	2.03 ± 1.19	1.92 ± 0.31	1.82 ± 0.43	2.16 ± 0.22

* *p* < 0.05, ** *p* < 0.01, compared with control group; the data represent the mean values ± SD.

**Table 8:** Selected absolute and relative organ weights induced by HZ1006 in rats following repeated dose administration after 28 days of continuous treatment and a 14-day recovery (only significant results are listed).

Organ	Days	Control	20 mg/kg	60 mg/kg	120 mg/kg
Absolute weight (female)					
Adrenal gland (g)	d28	0.068 ± 0.007	0.081 ± 0.005 **	0.068 ± 0.003	0.066 ± 0.008
	d42	0.077 ± 0.009	0.072 ± 0.008	0.071 ± 0.012	0.069 ± 0.011
Relative weight (female)					
Brain (g/100 g)	d28	0.799 ± 0.045	0.728 ± 0.047 *	0.799 ± 0.062	0.834 ± 0.042
	d42	0.739 ± 0.045	0.768 ± 0.020	0.768 ± 0.068	0.745 ± 0.089
Absolute weight (male)					
Testis (g)	d28	2.774 ± 0.209	2.707 ± 0.090	2.624 ± 0.170	2.666 ± 0.115
	d42	2.546 ± 0.082	2.798 ± 0.112 **	2.787 ± 0.097 **	2.723 ± 0.161 *
Relative weight (male)					
Kidney (g/100 g)	d28	0.759 ± 0.051	0.707 ± 0.024 *	0.705 ± 0.039 *	0.673 ± 0.023 **
	d42	0.705 ± 0.028	0.693 ± 0.033	0.687 ± 0.020	0.624 ± 0.053 **

* *p* < 0.05, ** *p* < 0.01, compared with control group; the data represent the mean values ± SD.

**Table 9:** Mean (± SD) toxicokinetic parameters of HZ1006 after oral administration in doses of 20, 60, and 120 mg/kg/day to SD rats.

Dose (mg/kg/day)	Duration of Administration	AUC_0–__∞_ (ng·h·mL^−1^)	C_max_ (ng·mL^−1^)	T_max_ (h)	t_1/2_ (h)
20 (*n* = 6)	single	8.0 ± 9.1	7.5 ± 7.2	0.86 ± 1.54	0.60 ± 0.37
	28 days	32.7 ± 14.9	12.9 ± 9.5	0.34 ± 0.18	7.32 ± 2.93
60 (*n* = 6)	single	39.1 ± 15.0	13.3 ± 5.5	0.50 ± 0.00	6.83 ± 2.69
	28 days	114.1 ± 30.9	19.7 ± 3.3	0.28 ± 0.17	7.57 ± 2.31
120 (*n* = 6)	single	69.9 ± 22.9	20.4 ± 3.8	0.45 ± 0.13	6.51 ± 1.62
	28 days	446.8 ± 282.2	57.5 ± 26.1	1.92 ± 3.07	7.03 ± 1.90
